# Quantitative Reevaluation of the Effects of Short- and Long-Term Removal of Descending Modulatory Inputs on the Pyloric Rhythm of the Crab, *Cancer borealis*^1^,^2^,^3^

**DOI:** 10.1523/ENEURO.0058-14.2015

**Published:** 2015-02-02

**Authors:** Albert W. Hamood, Sara A. Haddad, Adriane G. Otopalik, Philipp Rosenbaum, Eve Marder

**Affiliations:** Volen Center and Biology Department, Brandeis University, Waltham, Massachusetts 02545

**Keywords:** central pattern generator, homeostatic regulation of excitability, stomatogastric nervous system

## Abstract

The crustacean stomatogastric ganglion (STG) receives descending neuromodulatory inputs from three anterior ganglia: the paired commissural ganglia (CoGs), and the single esophageal ganglion (OG). In this paper, we provide the first detailed and quantitative analyses of the short- and long-term effects of removal of these descending inputs (decentralization) on the pyloric rhythm of the STG. Thirty minutes after decentralization, the mean frequency of the pyloric rhythm dropped from 1.20 Hz in control to 0.52 Hz. Whereas the relative phase of pyloric neuron activity was approximately constant across frequency in the controls, after decentralization this changed markedly. Nine control preparations kept for 5–6 d *in vitro* maintained pyloric rhythm frequencies close to their initial values. Nineteen decentralized preparations kept for 5–6 d dropped slightly in frequency from those seen at 30 min following decentralization, but then displayed stable activity over 6 d. Bouts of higher frequency activity were intermittently seen in both control and decentralized preparations, but the bouts began earlier and were more frequent in the decentralized preparations. Although the bouts may indicate that the removal of the modulatory inputs triggered changes in neuronal excitability, these changes did not produce obvious long-lasting changes in the frequency of the decentralized preparations.

## Abstract (precis)

Neuromodulatory inputs are known to strongly influence the intrinsic excitability of individual neurons and the networks in which the targets of modulation are found. It is therefore important to understand how nervous systems respond to altered neuromodulatory environments.

## Significance Statement

Neuromodulatory inputs are known to strongly influence the intrinsic excitability of individual neurons and the networks in which the targets of modulation are found. It is therefore important to understand how nervous systems respond to altered neuromodulatory environments. We find that removal of neuromodulatory inputs from the stomatogastric ganglion of wild-caught *Cancer borealis* decreases frequency and alters phase relationships of the pyloric rhythm within 30 min, and that these changes persist across time. Previous reports from several species suggested that the pyloric rhythm of crustaceans compensates for this loss and recovers intact activity patterns; our dataset suggests that this perturbation is not compensated. Caution is necessary when interpreting results from wild-caught populations, which may be unstable over time.

## Introduction

A fundamental but unresolved issue in neuroscience is how neurons and networks respond to the reduction or total loss of neuromodulatory inputs. The crustacean stomatogastric nervous system has been an extraordinarily useful preparation for the study of the fundamental mechanisms of central pattern generation, neuromodulation, and response to the removal of neuromodulatory inputs (Selverston et al., 1976; Selverston and Moulins, 1985, 1987; Harris-Warrick et al., 1992; Marder and Bucher, 2007). In the lobster, *Panulirus interruptus*, the importance of descending inputs from the paired commissural ganglia (CoGs) to the single stomatogastric ganglion (STG) for the generation of robust gastric mill and pyloric rhythms was first highlighted by reversible block of the stomatogastric nerve (stn) (Russell, 1976, 1979). This resulted in loss of gastric mill activity and decreases in the cycle frequency (subsequently referred to simply as frequency) of the pyloric rhythm, within minutes. Similar results have been subsequently obtained in a variety of crab and lobster species, although in some species, after removal of the descending inputs (decentralization), there may be a stronger tendency for the pyloric rhythm to fall silent (Moulins and Cournil, 1982; Bal et al., 1994).

The crab, *Cancer borealis*, has been used for a large number of studies of the effects of neuromodulators and neuromodulatory neurons (Nusbaum et al., 2001; Nusbaum and Beenhakker, 2002; Marder and Bucher, 2007; Blitz and Nusbaum, 2011, 2012). In *C. borealis*, there have been anecdotal reports that decentralization can result either in a decrease in the frequency of the pyloric rhythm, or its complete cessation. Because the effects of excitatory neuromodulators are more easily seen when the pyloric rhythm is either very slow or stopped, the specific examples used as figures demonstrating the effects of neuromodulators in the literature have been inadvertently biased towards preparations with less robust pyloric rhythms after decentralization. Consequently, this has fostered the perception that decentralization in *C. borealis* routinely results in the loss of all pyloric rhythm activity, although there have been no large data sets that address this issue. This has prompted us, in the first section of this paper, to provide data from more than a hundred preparations showing, for the first time, a quantitative analysis of the effects of short-term removal of the descending modulatory inputs. Quantitative data reporting the frequency and phase relationships of pyloric rhythms in *C. borealis* in the presence of the descending modulatory inputs were recently reported (Goaillard et al., 2009), and the control data in this study are largely consistent with those data.

Stomatogastric nervous system preparations can survive for many days after removal from the animal (Thoby-Brisson and Simmers, 1998, 2000, 2002; Golowasch et al., 1999b; Mizrahi et al., 2001; Luther et al., 2003; MacLean et al., 2003, 2005; Zhang et al., 2003). This creates the remarkable opportunity to use these preparations to study the effects of long-term removal of neuromodulation and the resultant decrease in activity on the circuits that are the direct targets of that neuromodulation.

It is now clear that extended periods of time deprived of neuromodulatory inputs trigger changes in excitability and gene expression (Mizrahi et al., 2001; Thoby-Brisson and Simmers, 2002; Temporal et al., 2012, 2014) in preparations from several species. There are previous reports that preparations that fall silent after decentralization recover rhythmic activity over several days (Thoby-Brisson and Simmers, 1998, 2000, 2002; Golowasch et al., 1999a; Luther et al., 2003), as would be expected if changes in excitability and gene expression that are triggered by removal of the descending inputs allow the STG to retune, using homeostatic or other rules to resume more normal function (Golowasch et al., 1999a; Luther et al., 2003; Zhang and Golowasch, 2007, 2011; Zhang et al., 2009;).

The increased computer power available today makes it possible to record continuously from preparations for days and weeks. We now report quantitative analyses of data from continuous recordings of STGs from *C. borealis* that show many features of the data previously reported and interpreted as recovery of normal pyloric activity. Nonetheless, these new data are more consistent with the interpretation that each animal maintains a relatively stable level of activity over several days after decentralization, although it is highly likely that numerous changes in system parameters are occurring during this time frame that, in principle, could support recovery of function in some preparations, had they lost rhythmic activity in a short time after decentralization.

## Materials and Methods

### Animals

Adult male *Cancer borealis* were purchased from Commercial Lobster and communally housed in artificial seawater at 10–12 °C until used, generally within 1 week of arrival. On the first day of the experiments, crabs were anesthetized by storing in ice for 30 min prior to the dissection. Animals used in this study were from May 2011 to November 2014, with most from 2013 and 2014.

### Saline composition

*C. borealis* physiological saline included 440 mM NaCl, 11 mM KCl, 13 mM CaCl_2_, 26 mM MgCl_2_, 11 mM Trizma base, and 5 mM Maleic acid. For long-term experiments, this saline was supplemented with 5.5 mM dextrose, 50 μg/ml penicillin/streptomycin (Gibco, Reference 15140-148), and 500 ng/ml fungizone (Amphotericin B, Omega Scientific, Catalog No. FG-70).

### In vitro long-term recordings

Petri dishes were disinfected by UV light for at least 30 min prior to use, and dissection tools were soaked in 70% ethanol. Intact stomatogastric nervous systems (STNS), including the STG, the bilateral commissural ganglia (CoGs), and the esophageal ganglion (OG) were dissected and pinned out on a Sylgard-coated Petri dish, and maintained in continuously superfused physiological saline containing antibiotics. Vaseline wells were built around the motor nerves containing the axons for the motor neurons of the pyloric rhythm, including the lateral ventricular nerve (lvn) and the pyloric dilator nerve (pdn). Preparations were then placed in a humidity-controlled environmental chamber and held at 12 °C, while fresh saline was continuously superfused at a rate of 300–500 ml per day. After recording 1 h of baseline data, decentralization was accomplished by transection of the stn approximately midway between the STG and the OG. In some cases (6/19), the severed anterior ganglia were removed from the dish following decentralization; this procedure did not qualitatively change any result. Dishes were then covered with parafilm and left otherwise undisturbed for the duration of the experiments while recordings continued. Fresh saline was pipetted into the Vaseline wells each day. This protocol reliably maintained a healthy STNS for long periods of time, lasting up to several weeks.

### Acute decentralization

After dissecting the STNS as described above, preparations were pinned out onto Sylgard-coated Petri dishes with Vaseline wells built around at least the lvn, and recordings were made during continuous superfusion of physiological saline at 10–12 °C. The superfusion rates often used in the acute experiments (1–10 ml/min) were considerably greater than those used in long-term experiments. Decentralization was accomplished by either transection of the stn, blockade of the stn with TTX (10^−7^ M), or both. No qualitative differences were observed as a result of the method of decentralization, nor the experimenter.

### Electrophysiological recordings

Data were acquired using a Digidata 1440 digitizer (Axon Instruments), a model 3500 extracellular amplifier (A-M Systems), and pClamp data acquisition software (Axon Instruments, version 10). Recordings always contained at least the pdn and lvn nerves, or a sufficiently clean recording of the lvn nerve alone that allowed for unambiguous detection of burst properties for the pyloric dilator (PD) and lateral pyloric (LP) units of the pyloric rhythm.

### Data analysis

Data were analyzed offline using custom scripts written in Spike2 (Cambridge Electronic Design, version 7) and Matlab (Mathworks, version 2013b). These scripts allowed for semi-automated detection of burst timing in PD and LP neurons. Phase relationships were quantified relative to PD start. For individual long-term plots of frequency against time, the median frequencies of 6 min (0.1 h) bins of pyloric data are plotted. For ANOVAs involving LP neurons, preparations in which LP was silent were not plotted as points in figures showing LP phase; pyloric rhythms lacking LP neuron activity were rare. Box plots show the median and first and third quartile of the given data, while whiskers extend to the most extreme datum within 1.5 times the interquartile range from the nearest quartile. Outliers are shown with an X.

### Statistics

Assessment of changes caused by decentralization was done by two-way, mixed-model ANOVA on averaged data binned across the first 6 d of recordings. Decentralization state was treated as a between-subjects factor, and time was treated as a within-subjects factor. Pairwise comparisons of groups were done by paired *t* tests when possible; unpaired, independent samples *t* tests were used when groups were not matched but had similar *n*'s and variances. Mood's median test was used for comparing groups of much different sizes and variances. Correlation *R* and *p* values are reported from linear Pearson correlations. All tests were computed in Matlab (Mathworks, version 2013b), and significance was assessed at the *p* < 0.05 level (Tables 1, 2, 3, 4).

**Table 1:** Pearson correlations

Data	*n*	*R*	*p* value
Intact, Hz vs PD off	123	0.4113	1.19E-05
Intact, Hz vs LP on	123	0.0555	0.5759
Intact, Hz vs LP off	123	0.3422	3.77E-04
Decentralized, Hz vs PD off	115	0.8229	<1E-06
Decentralized, Hz vs LP on	115	0.8298	<1E-06
Decentralized, Hz vs LP off	115	0.8295	<1E-06
Intact, Hz vs 30 min decentralized Hz	115	0.4077	6.1049E-06
30 min decentralized Hz vs 2 d decentralized Hz	19	0.5234	0.0258
2 d decentralized Hz vs 3 d decentralized Hz	19	0.6046	7.90E-03
3 d decentralized Hz vs 4 d decentralized Hz	19	0.4249	0.0788
5 h recovery time vs time to first bout	19	0.151	0.537

**Table 2:** *t* tests

Data	*n*	*t*	*p* value
Paired *t* tests			
Intact Pyloric Hz vs 30 min decentralized Hz	115	40.768	<1E-06
Hz, pre- vs post-saline exchanges (avg)	10	−5.2897	5.01E-04
Unpaired *t* tests			
Time-matched vs saline-exchange pre	13 matched; 10 exchanged	−2.4662	0.028
Time-matched vs saline-exchange post	13 matched; 10 exchanged	−5.2897	5.01E-04

**Table 3:** Mixed-model ANOVAs

Data	*n*	Between-subjects *F*	Between-subjects *p*	Within-subjects *F*	Within-subjects *p*	Interaction *F*	Interaction *p*
Pyloric Hz	19; 9	108.12	<1E-06	1.632	0.1578	1.775	0.1234
Cycle variation	19; 9	5.134	0.0347	0.426	0.82975	0.365	0.87119
PD off	19; 9	24.944	6.07E-05	0.254	0.93719	0.703	0.6226
LP on	19; 9	5.382	0.0489	0.687	0.63616	0.702	0.625
LP off	19; 9	9.234	0.016	2.175	0.076	0.55	0.7376
Bouts	19; 9	9.417	6.06E-03	7.337	6.8E-06	6.393	3.42E-06

**Table 4:** Mood's median tests

Data	*n*	*χ*^2^	*p* value
Fastest 5 h decentralized vs intact Pyloric Hz	19 5-h decentralized stretches; 123 intact	17.56	2.78E-05
Fastest 5 h decentralized vs intact cycle variation	19 5-h decentralized stretches; 123 intact	21.935	2.82E-06
Fastest 5 h decentralized vs intact PD off	19 5-h decentralized stretches; 123 intact	17.6187	2.70E-05
Fastest 5 h decentralized vs intact LP on	19 5-h decentralized stretches; 123 intact	8.1044	4.40E-03
Fastest 5 h decentralized vs intact LP off	19 5-h decentralized stretches; 123 intact	11.6703	6.35E-04
Fastest 5 h decentralized vs 30 min decentralized Hz	19 5-h decentralized stretches; 115 30-min decentralized	1.5332	0.2156
Fastest 5 h decentralized vs 30 min decentralized cycle variation	19 5-h decentralized stretches; 115 30-min decentralized	5.1989	0.0226
Fastest 5 h decentralized vs 30 min decentralized PD off	19 5-h decentralized stretches; 115 30-min decentralized	1.5585	0.2119
Fastest 5 h decentralized vs 30 min decentralized LP on	19 5-h decentralized stretches; 115 30-min decentralized	1.348	0.2456
Fastest 5 h decentralized vs 30 min decentralized LP off	19 5-h decentralized stretches; 115 30-min decentralized	1.348	0.2456

## Results

### Decentralization reduces cycle frequency and alters phase relationships in the pyloric rhythm

**Figure 1:**
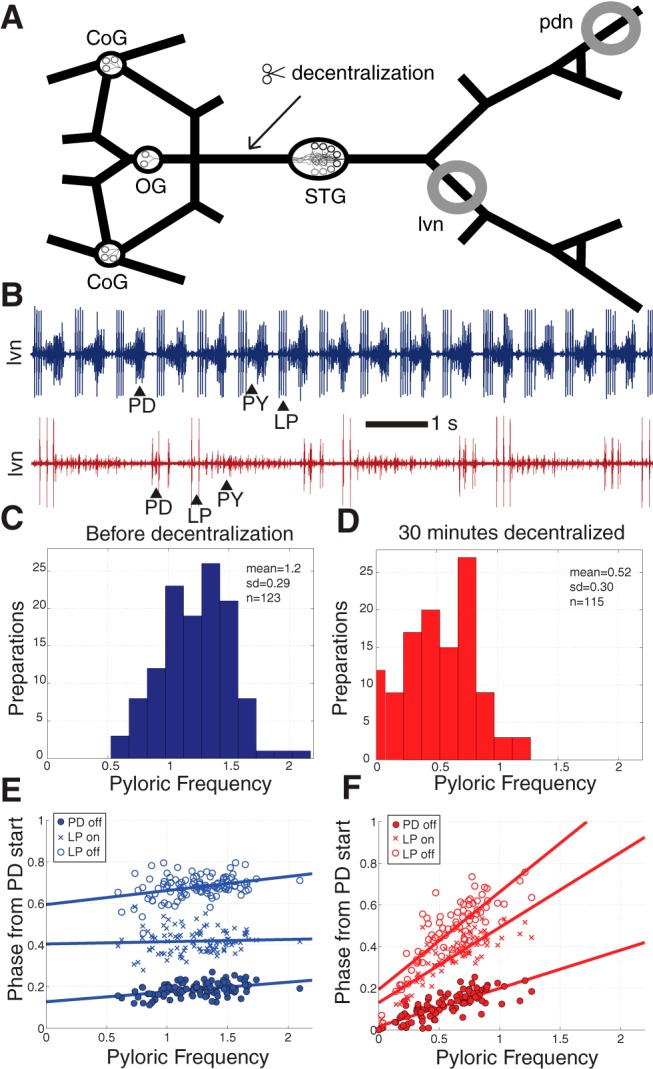
Acute effects of decentralization on the pyloric rhythm. ***A***, Schematic diagram of the dissected STNS, as pinned out for recording, showing the location of the STG and its associated anterior modulatory ganglia (CoG, OG). Grey circles identify where Vaseline wells were built for monitoring pyloric activity extracellularly. Decentralization was accomplished by blocking or transecting the stn at the indicated location. ***B***, Example lvn recordings from a preparation before (blue trace) and 30 min following decentralization (red trace). ***C, D***, Histograms of pyloric cycle frequencies immediately before decentralization (***C***, blue bars) and 30 min after (***D***, red bars). ***E, F***, Phase relationships among the PD and LP cells in the pyloric rhythm for intact (***E***, blue) and 30 min decentralized (***F***, red) preparations. Lines represent fits by linear regression.

Figure 1A shows a schematic diagram of the stomatogastric nervous system including the STG, which contains the circuit that drives the pyloric rhythm of the crab *C. borealis*. The rhythm consists of triphasic alternation between the bursting outputs of three components. The first component includes two PD neurons and a single anterior burster neuron (AB), which are electrically coupled and fire together, forming the pacemaker kernel of the network. Rhythmic inhibition from these neurons then drives bursting activity in follower neurons: a single LP neuron, which fires a burst of action potentials following the PD/AB burst; and a set of several pyloric constrictor (PY) neurons, which fire as the third component of the rhythm. The PD, LP, and PY neurons are motor neurons whose bursting activity drives rhythmic contractions of musculature in the stomach (Maynard, 1972; Maynard and Dando, 1974), and are also the core of the pattern-generating circuit, with many reciprocal inhibitory connections between them (Eisen and Marder, 1982). The axons of these neurons project through motor nerves that can be identified by their muscular targets, allowing activity in the network to be monitored extracellularly without disturbing somata within the STG.

Neuromodulatory inputs to the STG arrive through a single input nerve, the stn (Fig. 1A). Figure 1B shows a typical example of raw data recorded extracellularly from the lvn both before (blue trace) and shortly after (red trace) the acute removal of the effects of these modulatory inputs by decentralization. This preparation displayed a greatly reduced pyloric frequency following the perturbation, but maintained its characteristic triphasic rhythm. Consistent with previous reports, all preparations examined exhibited a reduced pyloric frequency following the removal of modulatory inputs.

Population frequency data are summarized in histograms in Figure 1C (intact preparations, blue) and Figure 1D (preparations decentralized for 30 min, red). Mean pyloric cycle frequency was significantly decreased following decentralization (1.2 ± 0.29 Hz before, 0.52 ± 0.30 Hz after, *p* < 0.001). However, a complete cessation of rhythmic activity was rare; less than 10% of preparations (9/115) displayed no rhythmic activity at 30 min post-decentralization. These data were collected by four different experimenters, over 2 years, and with variable protocols for decentralization, which involved either a TTX block of the stn, a complete transection of the input nerve, or both. Qualitatively, no differences in the effect of decentralization were observed as a result of any of these variables.

Phase relationships were also altered 30 min following decentralization (Fig. 1E,F). For intact preparations, pyloric phase was largely conserved across observed frequencies, consistent with previous reports (Bucher et al., 2005; Goaillard et al., 2009). However, this larger dataset did reveal small but statistically significant correlations between pyloric frequency and PD off-phase (*R* = 0.41, *p* < 0.001) and LP off-phase (*R* = 0.34, *p* < 0.001) (Fig. 1E). Interestingly, there was no correlation observed between pyloric frequency and LP on-phase (*R* = 0.055, *p* = 0.58), suggesting that this relationship may be a particularly important target of phase conservation in the pyloric rhythm.

Almost all previous studies that showed that the phase relationships of pyloric neuron activity were relatively independent of frequency across preparations were done on preparations with relatively high frequency rhythms, and with the anterior ganglia attached (Bucher et al., 2005; Goaillard et al., 2009; Tang et al., 2010). Following decentralization, pyloric phase relationships were less conserved, and stronger and steeper correlations were observed between pyloric frequency and PD off-phase (*R* = 0.82, *p* < 0.001) and LP off-phase (*R* = 0.83, *p* < 0.001). A strong correlation with frequency also emerged for LP on-phase (*R* = 0.83, *p* < 0.001) (Fig. 1F).

### Long-term recordings reveal varied activity following decentralization

**Figure 2:**
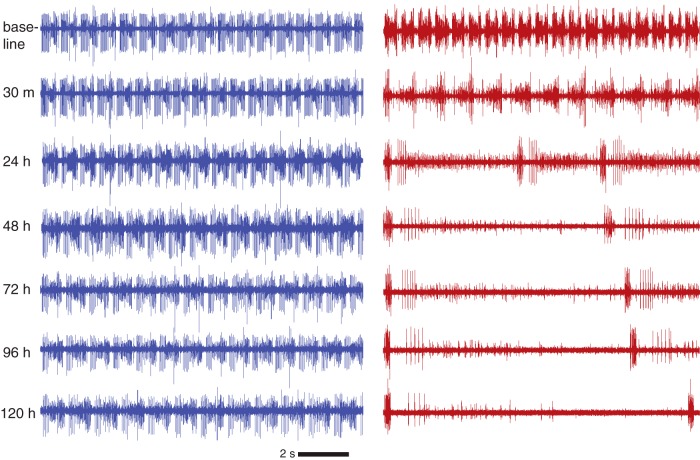
Long-term, continuous extracellular recordings of preparations of the STNS reveal stable behavior. Example 15 s lvn traces from two preparations, one in which with the stn left intact throughout the experiment (blue traces, left), and one in which the stn was transected shortly after the baseline recording (red traces, right).

Previous work on decentralized preparations of the STNS lacked quantitative descriptions, at the population level, of the rhythms generated continuously over many days. We maintained preparations of the STNS and recorded continuously for long durations lasting up to hundreds of hours (4–6 d or longer), superfusing saline throughout. We then compared the behavior of decentralized preparations across these long time-scales to that of intact, control preparations. Figure 2 shows example raw traces from the lvn of an intact preparation (blue traces, left) and a decentralized preparation (red traces, right) spanning the first 5 d in culture. This intact preparation maintained its rhythmic character and approximate cycle frequency throughout the experiment. Following decentralization, the preparation on the right displayed a slower frequency at 30 min, which was further reduced at 24 h. Subsequently, this preparation maintained a slower, more variable rhythm for the duration of the experiment.

**Figure 3:**
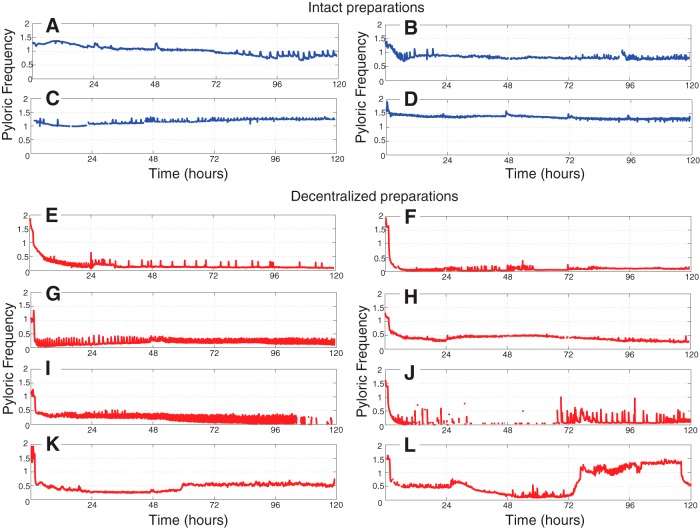
Example long-term pyloric cycle frequency plots of intact (***A−D***) and decentralized (***E−L***) preparations of the STNS. Data are plotted after binning frequencies into 0.1 h bins. Intact preparations (blue) tend to maintain stable pyloric rhythms with frequencies in the normal range of controls. Decentralized preparations (red) display more varied and variable behavior. Decentralization was accomplished approximately 1 h following the beginning of these recordings.

Figure 3 shows frequency against time plots for a number of examples of preparations maintained in this way, including the preparations shown in Figure 2 (intact example, Fig. 3A; decentralized example, Fig. 3E). Pyloric rhythms from intact preparations (blue traces, Fig. 3A−D) tended to maintain a frequency close to their original frequencies. Conversely, decentralized preparations (red traces, Fig. 3E−L) slowed rapidly following the removal of modulatory inputs, and then tended to approximately maintain this lowered frequency for the duration of the experiment. While intact preparations tended to behave similarly across animals and exhibit lower variation in their frequency output across time, decentralized rhythms were considerably more variable. Some preparations maintained a steady, lower-frequency rhythm throughout (Fig. 3G,H), while others exhibited wide variation in their frequency output on long time-scales (Fig. 3J−L) or short time-scales (Fig. 3E,I). While some preparations exhibited periods of complete silence (Fig. 3F, 3J), most had observable, slow pyloric rhythms at all times during these experiments. Excursions into the frequency range exhibited by intact preparations were rare (Fig. 3L, hours 75–115) and transient.

### Decentralized preparations do not recover intact-like pyloric rhythms

**Figure 4:**
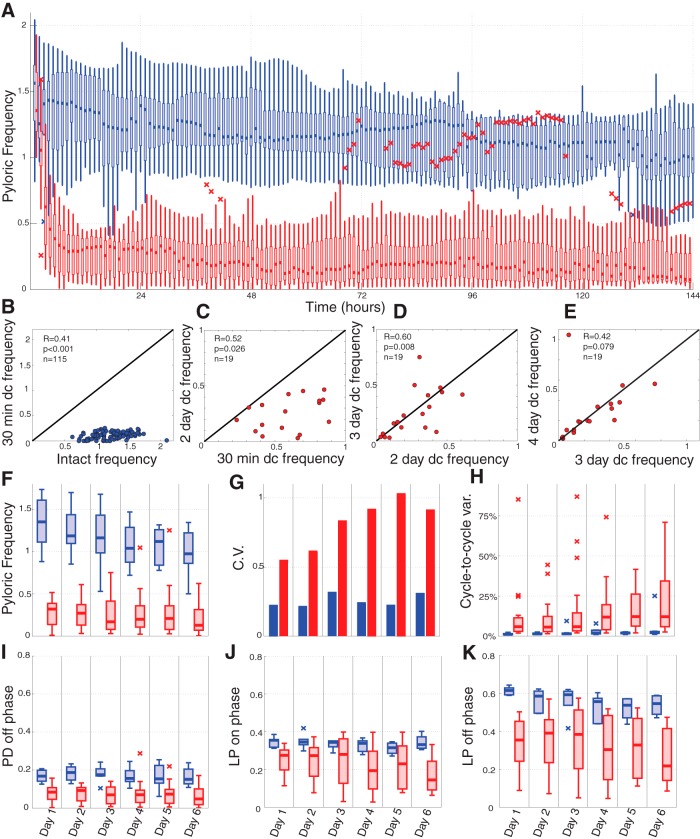
Long-term population effects of decentralization on the pyloric rhythm. ***A***, Population data for intact (*n* = 9) and decentralized (*n* = 19) preparations is shown for the first 6 d in culture. Data are plotted as box plots (see Materials and Methods) for each hour; data from intact preparations are shown in blue while decentralized preparations are shown in red. ***B−E***, Cycle frequency correlations within preparations across time (dc, decentralized). Black diagonal line represents unity. Insets report *R* and *p* values of Pearson correlations. ***B***, Following decentralization, all preparations exhibit a lower pyloric cycle frequency. ***C***, All examined preparations also exhibit a lower frequency on the second day following decentralization (average frequency, 24***−***48 h). ***D, E***, On subsequent days, preparations are more correlated, and show an equal tendency to increase or decrease frequency. ***F−K***, Measures of frequency (***F***), across-preparation variability (coefficient of variation (C.V.), ***G***), within-preparation variability (***H***), and pyloric phase relationships (***I−K***) are plotted for intact (blue) and decentralized (red) preparations. Box plots are created from population data consisting of the average value for each preparation in 24 h bins following decentralization.

Figure 4 summarizes population data for the pyloric rhythm in long-term intact (blue data) and decentralized (red data) preparations of the STNS. Figure 4A shows hourly box plots of pyloric cycle frequency for intact preparations (blue, *n* = 9) and decentralized preparations (red, *n* = 19) extending through the first 6 d. These groups rapidly diverged following decentralization, and remained so throughout, with little change through time in the frequency distributions for either group. Little overlap between the frequency ranges was observed, although one decentralized outlier produced a fast rhythm for many hours (see also Fig. 3L).

To assess the degree to which individual preparations varied in their frequency across time, we plotted correlations in pyloric frequencies across time (Fig. 4B−E). This also allowed us to address the hypothesis that slower preparations, which fall silent or near-silent, will better engage compensatory mechanisms and recover faster rhythms than those that do not lose their rhythmic character following decentralization. From immediately before decentralization to 30 min following decentralization, a significant positive correlation was observed (Fig. 4B, *p* < 0.001), indicating that faster intact rhythms tended to also be among the faster decentralized rhythms. All preparations were markedly slower, as can be observed by comparison to the unity line. However, as measured by the correlation *R* value, these correlations were stronger at later time-points following decentralization. When comparing frequencies at 30 min following decentralization to the average frequency observed during the second day (hours 24–48, Fig. 4C), a significant correlation was observed (*p* < 0.0258) and all preparations again exhibited slower rhythms. Thus, the slow-down process is not yet complete at 30 min following decentralization. Nonetheless, relatively few of these preparations exhibited a complete loss of pyloric rhythmicity. On subsequent days, pyloric frequency was highly correlated within preparations, and preparations were equally likely to increase or decrease frequency from one day to the next (Fig. 4D,E). Thus, we found no evidence that preparations that become slower recover more effectively to faster frequencies; preparations that became very slow following decentralization tended to remain so on subsequent days.

We next quantified the degree to which features of the pyloric rhythm changed over time and as a result of decentralization to look for quantitative evidence of recovery at the population level. We binned pyloric data within preparations by day and assessed the observed changes by a two-way, mixed-model ANOVA (see Materials and Methods, above). Decentralization significantly decreased pyloric cycle frequency (Fig. 4F, *p* < 0.001), PD off-phase (Fig 4I, *p* < 0.001), LP on-phase (Fig. 4J, *p* = 0.048), and LP off-phase (Fig. 4K, *p* = 0.016), and significantly increased variability both within preparations (Fig. 4H, *p* = 0.035) and across preparations, as measured by the coefficient of variation (Fig. 4G). However, there was no significant effect of time on any of these features, nor was there any significant interaction between time and modulatory condition. Decentralization produced quantitative changes in the pyloric rhythm that did not decrease for as long as these experiments were run, suggesting that the rapid changes induced by decentralization were not compensated for within the conditions of the experiment.

### Bouting is increased following decentralization, and increases over time

Previous work on decentralization in the STG has described bouting, a phenomenon in which decentralized preparations experience transient increases in pyloric frequency (Golowasch et al., 1999b; Luther et al., 2003; Zhang et al., 2009), which were thought to precede a stable recovery. Indeed, the time to first observed bout has been used as a proxy for recovery (Zhang et al., 2009).

To quantify the bouts seen in our long-term recordings, we applied the previously used definition for bouts (Luther et al., 2003), in which a bout was defined as three or more pyloric cycles with a >40% increase relative to the background frequency. We computed background frequency as a trailing average of 10 pyloric cycles, and compared it to the following three pyloric cycles. A bout was recorded if all three of the following cycles exhibited at least a 40% increase from the mean of the previous ten.

**Figure 5:**
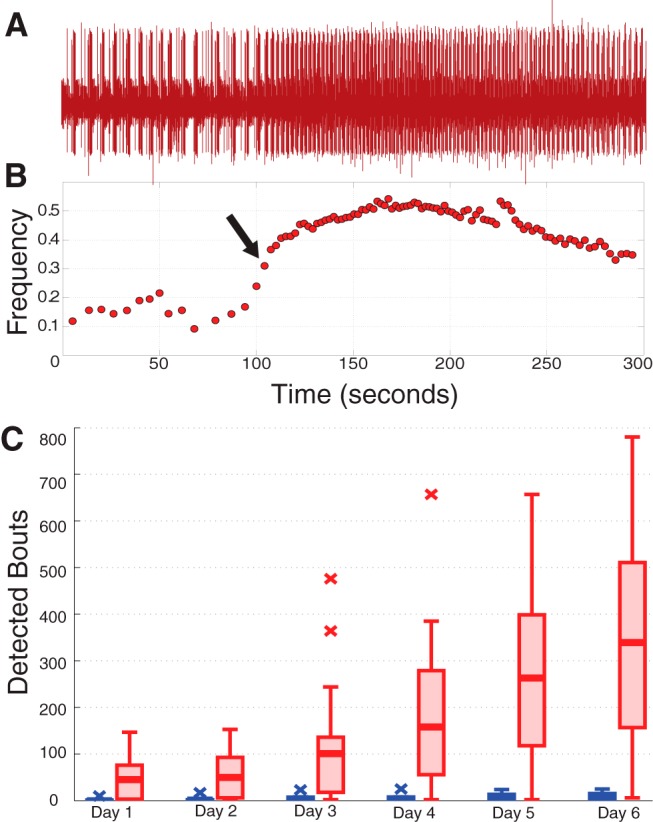
Analysis of bouting. ***A***, Example lvn recording featuring one detected bout, visible here in the noticeable increase in pyloric cycle frequency starting mid-trace. ***B***, Frequency plot corresponding to the data in ***A***. Arrow designates the point at which the bout was detected. ***C***, Box plots show population data of the number of detected bouts by day for intact (blue) and decentralized (red) preparations.

Figure 5A shows a 5 min stretch of raw data from the lvn of a decentralized preparation containing a frequency bout. The corresponding frequency versus time plot is shown below (Fig. 5B) and the arrow represents the point at which this bout was detected. Summary population data for bouting are shown in Figure 5C. As previously reported, decentralized preparations displayed bouting behavior, and did so significantly more than intact preparations (Fig. 5C, *p* = 0.006, ANOVA). However, rather than presaging recovery to a stable, intact-like pyloric rhythm, we found that bouting increased over time, both for intact and decentralized preparations (*p* < 0.001, ANOVA). Further, a significant interaction was observed between modulatory state and time (*p* < 0.001), as decentralized preparations tended to experience greater increases in bouting over time than did intact preparations. These observations are consistent with the hypothesis that bouting is indicative of a retuning process engaged by decentralization, which may then further increase over time.

### Previous definitions of recovery yield results similar to acute decentralization

**Figure 6:**
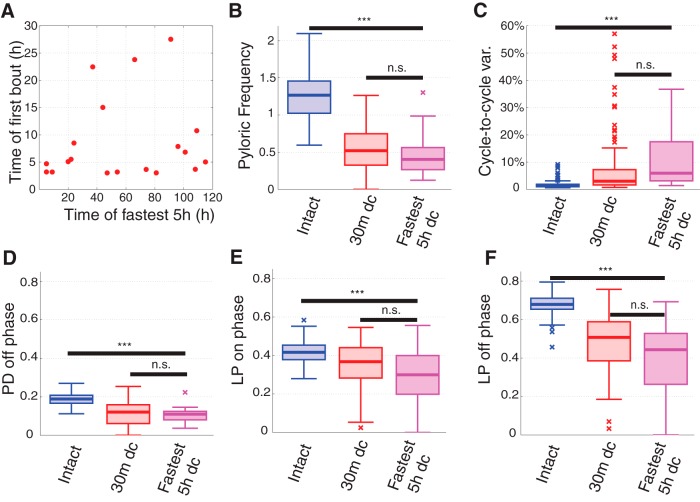
Analysis of previously used measures of recovery. ***A*. **Two previously described definitions of recovery (time to first bout, and 5 h strong and steady rhythm) fail to correlate. ***B−F***, Box plots show population data of intact (blue), 30 min decentralized (dc; red), and the fastest 5 h post-decentralization period of data from each decentralized preparation within the first 120 h. n.s., not significant; ***p < 0.001.

The earliest reports of recovery after decentralization (Thoby-Brisson and Simmers, 1998, 2000, 2002; Golowasch et al., 1999b) showed convincing examples of recovery, but little indication of how representative these examples were of the population. Subsequently, two definitions of recovery have also been used. Luther et al. (2003) defined recovery as a “strong and stable pyloric rhythm that lasted at least five hours”. While the authors did not report population data regarding when these recoveries occurred or the frequencies typically observed, they did report that virtually all preparations met this definition at some point. Thus, to implement an examination of this definition, we selected the fastest 5 h stretch of decentralized data for each preparation, and quantified features of pyloric rhythms observed during these periods. When we compared the fastest 5 h stretches of pyloric data in the long-term recordings to acutely decentralized preparations of the STNS, we found that the observed rhythms were nearly indistinguishable from those observed at 30 min following decentralization. No significant differences were observed between these 5 h periods and acutely decentralized pyloric rhythms for pyloric cycle frequency, PD off-phase, LP on-phase, or LP off-phase. However, these 5 h stretches did show significantly more cycle-to-cycle variability than that of acutely decentralized preparations (*p* = 0.023), consistent with our observation that bouting increases over time in long-term preparations (see Fig. 5). However, significant differences were observed between the 5 h stretches and rhythms produced by intact preparations for pyloric frequency (Fig. 6B, *p* < 0.001), cycle-to-cycle variability (Fig. 6C, *p* < 0.001), PD off-phase (Fig. 6D, *p* < 0.001), LP on-phase (Fig. 6E, *p* = 0.004), and LP off-phase (Fig. 6F, *p* < 0.001). Thus, the 5 h “fast stretches” resemble more closely those following decentralization than they do preparations that have not been decentralized.

A second definition of recovery applied in previous literature is the time to first bout (Zhang and Golowasch, 2007, 2011; Zhang et al., 2009). We found no correlation between the time to first bout and the time of onset of the fastest 5 h stretch (Fig. 6A, *p* = 0.537), suggesting that these two definitions do not measure the same thing, although both are likely to be indicative that changes in excitability are ongoing in the decentralized preparations.

### Regular saline exchanges excite the pyloric rhythm

**Figure 7:**
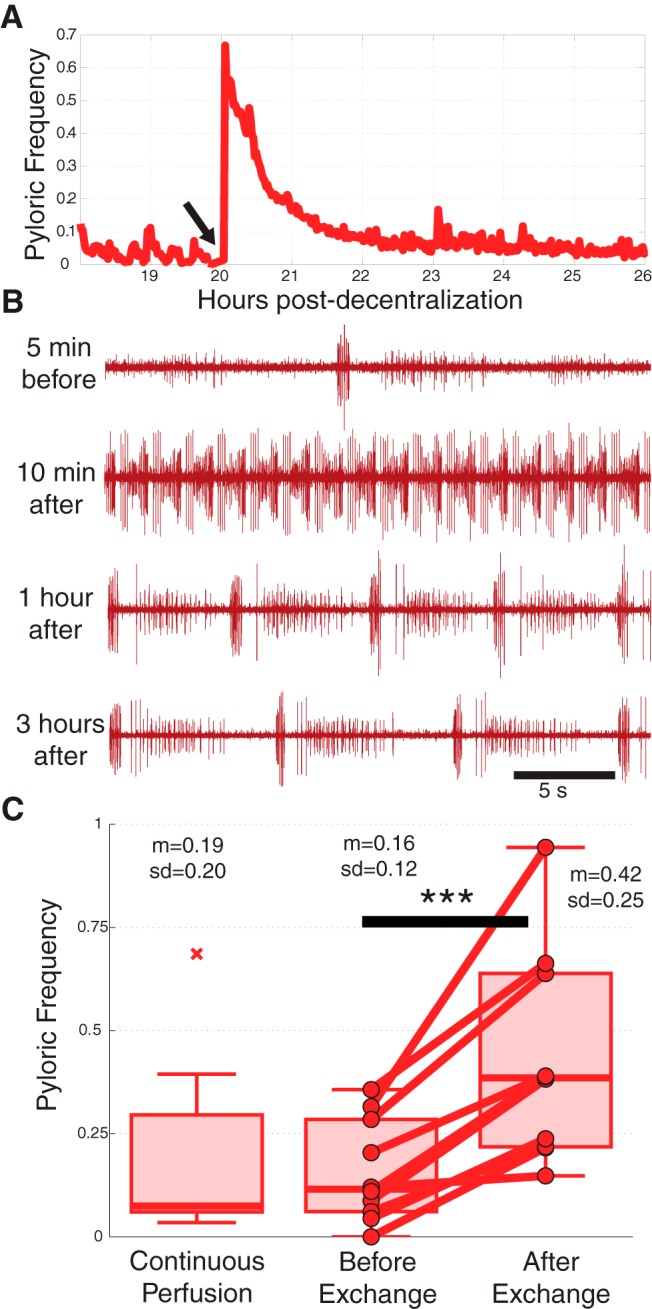
Daily saline exchanges transiently activate the pyloric rhythm. ***A***, Example pyloric cycle frequency plot for an 8 h stretch surrounding a saline exchange that transiently activates the pyloric rhythm (done at arrow). ***B***, Raw data from the same preparation reveals an increased frequency lasting several hours. ***C***, Population data showing the average effect of exchanging saline in 10 long-term decentralized preparations. Left-most box shows time-matched data from continuously superfused preparations. ***p < 0.001.

In our first experiments, we placed the preparations in a temperature-controlled sterile incubator and, instead of continuous superfusion, we changed the saline once per day. These preliminary experiments showed that the act of exchanging saline itself can activate the pyloric rhythm. We demonstrated this directly in a population of 10 long-term decentralized preparations in which saline was exchanged at least once per day by removal of the dish from the incubator and replacement with fresh, temperature-matched saline. Figure 7A shows an example frequency versus time plot for an experiment in which the first daily saline exchange produced a long-lasting activation of the pyloric rhythm (Fig. 7B). We quantified the extent to which preparations were affected by this procedure by averaging pyloric frequencies for each preparation in the 1 h before and the 1 h following saline exchanges. Saline exchanges, on average, increased the frequency for all preparations examined (Fig. 7C, *p* < 0.001). We compared the frequencies observed in this population to those of time-matched stretches of data from continuously superfused preparations and found that continuously superfused frequencies were significantly lower than frequencies shortly following saline exchanges (*p* = 0.02), but not significantly different than frequencies prior to saline exchanges.

## Discussion

In the early days of neuroscience, many fundamental discoveries and observations were reported with no indication of their reliability, although presumably the careful scientists of those days repeated experiments enough times to convince themselves of the reproducibility of their observations. For example, in the first paper that reports the results of the removal of the descending modulatory inputs to the STG of the lobster, *Panulirus interruptus*, Russell (1979) presented exquisite intracellular recordings showing the change in the waveforms of several pyloric rhythm neurons, but there are no *n*’s or statistics reported in the paper. In these early days, data were often collected by filming the oscilloscope screen, so it was routine to only have a snapshot or a stretch of data for later analysis. Moreover, in the absence of digital computers, data analysis meant measuring with rulers, or counting spikes or bursts by hand, again making it difficult to analyze long-stretches of data, even had it been reasonable to collect it.

We have undergone a complete transformation in the way in which we collect, store, and analyze data, and it is to be expected that more complete analysis of much larger data sets will force some modifications of what we learned from the earlier workers, although a remarkably large number of early observations are validated by later studies.

Additional complications have arisen that may require reevaluations or reinterpretations of the conclusions of already published work. For example, work on the stomatogastric nervous system has been performed on more than 10 different species of decapod crustaceans, including lobsters, crabs, crayfish, and shrimp. While almost all of the big-picture general principles that have come from work on the stomatogastic nervous system are revealed in all of these species, each species has its own idiosyncratic properties, and it has been one of our challenges to exploit these differences while not forgetting that they exist. This is relevant to the present study because the work on recovery of function subsequent to decentralization has been done on several species.

A thornier issue arises because we routinely study adult wild-caught animals (by carapace width, approximately 5–7 inches) that have lived in the ocean for multiple years before they are caught and sold to commercial distributors. They are then held in running sea-water tanks for an indeterminate period of time before they arrive in our laboratories. Consequently, we have no idea if we are sampling the same population of animals over time, or when the animals themselves are modified by a changing local environment. For example, after a particularly warm winter, the pyloric rhythms in preparations of *C. borealis* were far more robust in response to high temperatures than preparations from several prior years (Marder et al., 2015). Therefore, comparisons of data collected recently with those collected many years ago may be complicated by the non-stationarity of the animal population. That said, none of the previous studies on the effects of long-term decentralization had the statistical power and continuous recordings of the data presented here.

### Variability in response to acute decentralization

The control data presented here (Fig. 1) before removal of the descending inputs are similar to those previously published (Goaillard et al., 2009), albeit collected 5–7 years apart by different investigators. The two data sets have similar means and also show a wide range of starting frequencies, with the range being slightly larger in the data shown here, which may not be surprising since the *n* is almost twice as large.

Despite the large number of papers that have used decentralization to decrease the activity of STG preparations, this paper shows the first quantitative description of the effects of decentralization on the cycle frequency and phases of the pyloric rhythm. The population response to decentralization was robust, and every individual preparation slowed in response to decentralization. That said, only about 10% of the preparations actually stopped entirely after 30 min of removal of the descending modulatory inputs, and approximately the same number showed frequencies more characteristic of preparations with the descending inputs intact (Fig. 1D).

Another new observation made in this study is the difference in the phase of the LP neuron’s activity under the two conditions. Phase maintenance of the pyloric neurons across frequency has been viewed as one of the hallmarks of the pyloric rhythm (Hooper, 1997; Manor et al., 2003; Bucher et al., 2005; Mouser et al., 2008; Goaillard et al., 2009; Tang et al., 2010), and our data resemble closely those of previous studies for the control, modulatory inputs intact condition (Fig. 1E). Nonetheless, in the decentralized case, the phase is not constant as frequency is varied, but instead is advanced at lower frequencies. This is easy to understand as the pacemaker burst cannot maintain constant duty cycle at arbitrarily slow frequencies. It is interesting that the transition from phase compensated to non-compensated comes around the low end of the frequencies seen in the intact preparations. This frequency-dependent distinction between phase-compensated and non-phase-compensated pyloric rhythms has not been previously shown.

### Within-preparation stability and across preparation variability in long-term recordings

We maintained STGs for many days after dissection while slowly superfusing with physiological saline. When the descending modulatory inputs were left intact, each preparation maintained a characteristic average frequency that was within the distribution of frequencies seen in freshly dissected preparations. Likewise, decentralized preparations also maintained an approximately characteristic average frequency that again was within the distribution of frequencies seen in freshly dissected and acutely decentralized preparations. That said, there was considerably more jitter in the behavior of the decentralized preparations, and there were examples of extended periods of time when an individual preparation either sped up or slowed down. Had we not been recording continuously for many days, but sampling intermittently, some of these frequency changes might have looked as if the preparations were recovering from the effects of decentralization. Another possible confound is the effect of changing saline, which elicits several hours of higher frequency, normal-looking pyloric rhythms (Fig. 7). There are many potential factors that could contribute to this observation. Mechanical stimulation from the saline exchange operation may excite neurons directly, or cause neuromodulators to be released from the severed axon terminals of the stn. Temperature increases caused by prolonged opening of the incubator, or movement of the preparation out of the incubator could cause an increase in frequency. A depletion of dextrose or oxygen may slow the rhythm over time and leave it primed to increase rapidly following replenishment. A build-up of some unknown metabolic factor near the neurons of the STG, carried away regularly under conditions of continuous superfusion, could be responsible. It is unclear whether any of the previous reports describing recovery of function benefitted from this kind of stimulation by saline changes.

### Long-term changes in excitability

It is without doubt that changing the modulatory environment and activity of neurons, either in isolation or in circuits, evokes changes in excitability (Turrigiano et al., 1994, 1995; Thoby-Brisson and Simmers, 1998, 2000, 2002; Desai et al., 1999; Golowasch et al., 1999a; Cudmore and Turrigiano, 2004; O'Leary et al., 2010; Temporal et al., 2014), and these kinds of excitability changes may be important for eventually understanding regulation of neuronal excitability after events such as spinal cord damage or other large lesions. For this reason, the idea of complete recovery of function after removal of descending modulatory inputs is extremely attractive. Nonetheless, whether or not full recovery occurs routinely, it is plausible that many changes in channel expression are triggered by a dramatic loss of descending modulatory input and consequent decreases in activity (Khorkova and Golowasch, 2007; Temporal et al., 2014). Because there are so many neuromodulators released into the neuropil of the STG (Billimoria et al., 2005; Marder and Bucher, 2007; Cape et al., 2008; Blitz and Nusbaum, 2011; Marder, 2012), and their effects are found on every neuron of the STG (Swensen and Marder, 2000, 2001; Marder and Bucher, 2007; Harris-Warrick and Johnson, 2010), even if loss of neuromodulatory control triggers a series of compensatory changes, these homeostatic changes (O'Leary et al., 2013, 2014) might be slow to occur, or might require a significant period of time of more complete inactivity to be fully elicited. The bouts that were seen here and in previous studies may be indications of an ongoing network retuning process (Luther et al., 2003; Zhang and Golowasch, 2007, 2011), which was triggered more rapidly by the decentralization but to a lesser extent by removal from the animal, with its sensory and hormonal inputs.

One of the features of the previous reports of pyloric rhythm recovery is that the frequencies of the recovered rhythms were always relatively slow, in the range of those we recorded in both the acute and long-term conditions. It would be interesting to see if more pronounced recoveries of pyloric rhythms occur in a large group of animals that fall silent when the neuromodulatory inputs are acutely removed.

As we enter the era of “big data”, it is clear that those of us who work on wild-caught animals should adopt more strict standards of reporting, to the extent to which we are able, of the dates and sources of the animals we use for each study. In so doing, future generations of scientists may find it easier to find patterns in the variability we see among the individuals in these wild-caught animals.

## Synthesis

The decision was a result of the Reviewing Editor Marlene Bartos and the peer reviewers coming together and discussing their recommendations until a consensus was reached. A fact-based synthesis statement explaining their decision and outlining what is needed to prepare a revision is listed below. The following reviewers agreed to reveal their identity: Michael Nusbaum

### Reviewer 1:

GENERAL COMMENTS: This manuscript provides several important contributions to studies of neural circuit regulation. For example, it provides a firmer quantitative foundation for future studies of the short- and long-term consequences of removing modulatory input to a well-defined neural circuit. Second, its novel emphasis on the importance of documenting the details of the source and maintenance of each animal used in a study involving wild-caught animals is advice that should be followed by all investigators using such animals. While there will likely never be sufficient information available to fully establish the "pedigree" of each individual, the ability to catalog known variables could well help account for some amount of data variability or wholesale changes that take place across time. This manuscript should be of interest to the broad readership of eNeuro.

SPECIFIC COMMENTS- MAJOR:

1. None.

SPECIFIC COMMENTS- MINOR:

1. Title: Add a hyphen after "short".

2. Line 31: Change "markedly changed" to "changed markedly".

3. Line 32: (a) Change "pyloric rhythms" to "pyloric rhythm".

(b) Change "19" to "Nineteen".

4. Line 47: Change to "decreases frequency and alters phase relationships of the pyloric rhythm within 30 minutes,".

5. Lines 48-50: I hope this is not confusing, but I am not certain that the sentence that starts on Line 48 and ends on Line 50 (".... the pyloric rhythm of crustaceans compensates for this loss and recovers intact activity patterns; our dataset suggests that this perturbation is not fully compensated") accurately reflects the features that most clearly distinguish the previous and current studies of this issue. If this is indeed the case, then please modify this sentence.

(a) After just reading the Luther et al. (2003, J Neurophysiol) paper, it seems that this earlier work showed partial compensation/recovery (e.g. in some experiments, compensation occurred by recovering the rhythmic pattern which had turned off immediately after decentralization, but in general the cycle frequency remained slower than pre-decentralization; in other experiments, compensation was evident from the rhythm being very slow immediately after decentralization to partial recovery [∼0.5 Hz] of normal rhythm speed [∼1 Hz] after several days).

(b) It seems that, in the current manuscript, there is less emphasis on whether or not there was recovery from silence (because it did not happen often; <10% experiments). Instead the focus is on whether there is recovery from an initially slow cycle frequency post-decentralization. The finding is that, while the average final cycle frequency post-decentralization is the same as in Luther et al. (2003), in this manuscript there is little or no compensation/recovery of cycle frequency from the 30 min post-decentralization time-point to the day 4 time-point. This difference is suggested to result from the technical improvements in data acquisition and analysis that enabled continuous recording of these rhythms and a more extensive analysis of them. While I am confident that the latter is the case, upon just reading Luther et al. (2003) I learned that the pyloric rhythms were continuously recorded across many days in culture in that study as well.

6. Lines 119-120: Please subscript the "2" in "CaCl2" and "MgCl2", and add the pH of the saline.

7. Line 131: Please use a different word than "primary" to describe the key motor neurons of the pyloric rhythm, because in some systems (e.g. zebrafish) the phrase "primary motor neurons" is used to distinguish those neurons from the "secondary motor neurons" which innervate the same muscles but have different synaptic response and intrinsic properties.

8. Line 134/Line 144: These nervous systems were "superfused at a rate of 300-500 ml per day (∼0.2 - 0.4 ml/min)". How does its per minute rate compare to the per minute superfusion rate used during acute experiments?

9. Line 197: Insert "The first component includes" to the start of the sentence that currently begins with "Two PD neurons".

10. Line 200: Perhaps "primes activity" is better than "drives activity"?

11. Line 204: Add a comma after "pattern generating circuit".

12. Line 209: Add "(Fig. 1A)" after "single input nerve, the stn".

13. Line 222: Add "either" after "which involved".

14. Line 259: Delete "from".

15. Line 281: (a) add "positive" between "significant" and "correlation"? (b) The R-value is not provided in the figure; add it here in the text?

16. Line 284: Another mention of the use of R-values which themselves are not provided in either the text, figure or figure legends?

17. Line 318: Insert "at least" after "cycles exhibited".

18. Line 365: Change to "incubator, and instead of continuous superfusion we changed the saline once per day."

19. Lines 374-377: I think that there is an inaccuracy here- Based on Figure 7, it appears that the mean pyloric cycle frequency post-bulk saline exchange is not different from that occurring during continuous perfusion but is significantly different (p<0.001) from that occurring immediately before the bulk exchange. The current text appears to report instead a difference between continuous perfusion and post-bulk exchange but no different pre- vs post-bulk exchange.

20. Line 407: How were the crabs determined to be between 5-7 years old?

21. Lines 410-411: Change "In fact" to "For example".

22. Line 411: Insert "the pyloric rhythm in" after "a particularly warm winter,".

23. Lines 433-435: Merge references within the same parentheses.

24. Lines 438-439: Why is this point interesting?

25. Line 439-440: Has this cycle frequency-dependent distinction between phase-compensated vs not phase-compensated published previously?

26. Lines 443-456: What is suggested by the result that cycle frequency increased after bulk saline replacement? That the circuit remains largely unchanged from the control circuit but has a weaker drive due to reduced pacemaker activity caused by oxygen/nutrient starvation, and that the resulting weaker rhythm drives activity-dependent changes to occur in the circuit neurons? Something else?

27. Figures- Nerve Recordings:

(a) It would be helpful to have the extracellular nerve recordings labeled with the acronym of the recorded nerve so that the reader would not need to refer to the figure legend.

(b) It would be helpful if each nerve recording in a set (i.e. recordings at different time points) started at the same time point of the rhythm (e.g. a PD neuron burst). Doing so would make it much easier to compare rhythm speed across time points.

28. Figure 4B-E: Please define "dc" in the Figure Legend (dc, decentralization).

### Reviewer 2:

Although many previous studies have examined the activity of the pyloric circuit after decentralization, this study takes a very thorough quantitative approach with high n values and importantly utilizes continuous long-term recordings instead of sampling at discrete time points. This is a very careful study, the data are novel and very clearly presented and important findings are reported.

The data in this study alter our view of the consequences of removing modulatory inputs. Previous studies suggested cessation of circuit activity that recovered in a periodic fashion (bouting) until arriving at a new solution. This study suggests instead that the more common consequence of removal of modulatory input is a new "normal" which is arrived at much more quickly and then maintained. Interestingly, the new solution has both similarities and differences to circuit output in the presence of modulatory inputs. The authors also point that that comparisons of these data to previous data sets, to the extent possible, highlight complications that can arise from using wild-caught research animals, an important note for the scientific community. Additionally, comparing different measures used in previous studies to define "recovery" illustrated that these are actually not measuring the same thing which is important that the field be made aware of this for future consideration of both previous and future data. I have only minor comments.

Minor comments:

1. lines 261-263. Figure 3G is apparently mis-referenced as being both an example of a steady, lower-frequency rhythm throughout as well as exhibiting wide variation on short-time scales.

2. Line 350. I believe this should actually be referencing Fig. 4 which contains the plots of CV and cycle to cycle variability.

3. Lines376-378. "....post-exchange frequencies were significantly increased relative to continuously superfused preparations, but not significantly different than frequencies prior to saline exchanges." Aren't the post-exchange frequencies in fact different from those prior to saline exchanges?

4. Line 433. Include Tang et al 2010 within same parenthesis set as the rest of the references which follow.

5. Line 638. Delete "after" at the end of the line.

6. Line 663. There is no arrow in (A), only in (B).

7. Line 677. Delete "to".

## Author Response

GENERAL COMMENTS: This manuscript provides several important contributions to studies of neural circuit regulation. For example, it provides a firmer quantitative foundation for future studies of the short- and long-term consequences of removing modulatory input to a well-defined neural circuit. Second, its novel emphasis on the importance of documenting the details of the source and maintenance of each animal used in a study involving wild-caught animals is advice that should be followed by all investigators using such animals. While there will likely never be sufficient information available to fully establish the "pedigree" of each individual, the ability to catalog known variables could well help account for some amount of data variability or wholesale changes that take place across time. This manuscript should be of interest to the broad readership of eNeuro.

SPECIFIC COMMENTS- MAJOR:

1. None.

SPECIFIC COMMENTS- MINOR:

1. Title: Add a hyphen after "short". Done

2. Line 31: Change "markedly changed" to "changed markedly". Done

3. Line 32: (a) Change "pyloric rhythms" to "pyloric rhythm".

(b) Change "19" to "Nineteen". Done

4. Line 47: Change to "decreases frequency and alters phase relationships of the pyloric rhythm within 30 minutes,". Done

5. Lines 48-50: I hope this is not confusing, but I am not certain that the sentence that starts on Line 48 and ends on Line 50 (".... the pyloric rhythm of crustaceans compensates for this loss and recovers intact activity patterns; our dataset suggests that this perturbation is not fully compensated") accurately reflects the features that most clearly distinguish the previous and current studies of this issue. If this is indeed the case, then please modify this sentence.

(a) After just reading the Luther et al. (2003, J Neurophysiol) paper, it seems that this earlier work showed partial compensation/recovery (e.g. in some experiments, compensation occurred by recovering the rhythmic pattern which had turned off immediately after decentralization, but in general the cycle frequency remained slower than pre-decentralization; in other experiments, compensation was evident from the rhythm being very slow immediately after decentralization to partial recovery [∼0.5 Hz] of normal rhythm speed [∼1 Hz] after several days).

The Luther et al. (2003) paper does not show long-term recorded intact preparations; therefore it may be considered consistent with previous reports such as Thoby-Brisson and Simmers (1998) in which a small number of intact preparations slowed down after several days in culture. Thus, while the “recovered” examples in Luther et al. are indeed slower than pre-decentralization, they may be interpreted as similar to an intact pattern held for long-term. There is also an analysis of phase in at least one example preparation in Luther et al. suggesting that recovered phase relationships are restored to those of intact preparations.

While there is a lot of variability in the details of what has been reported in previous work, we believe the general consensus of those papers, as has been described in introductions, discussions, and review articles, is reflected in the sentence as written. The lack of a consistent definition of recovery makes it difficult to accurately and briefly summarize all the previous reports. Removed the word “fully” to help clarify.

(b) It seems that, in the current manuscript, there is less emphasis on whether or not there was recovery from silence (because it did not happen often; <10% experiments). Instead the focus is on whether there is recovery from an initially slow cycle frequency post-decentralization. The finding is that, while the average final cycle frequency post-decentralization is the same as in Luther et al. (2003), in this manuscript there is little or no compensation/recovery of cycle frequency from the 30 min post-decentralization time-point to the day 4 time-point. This difference is suggested to result from the technical improvements in data acquisition and analysis that enabled continuous recording of these rhythms and a more extensive analysis of them. While I am confident that the latter is the case, upon just reading Luther et al. (2003) I learned that the pyloric rhythms were continuously recorded across many days in culture in that study as well.

Luther et al. (2003) did continuously record, but they did not continuously superfuse saline. They also did not quantitively analyze those long-term data (it’s quite a big job even on our modern computers), so it is difficult to compare our results directly with theirs.

6. Lines 119-120: Please subscript the "2" in "CaCl2" and "MgCl2", and add the pH of the saline. Done

7. Line 131: Please use a different word than "primary" to describe the key motor neurons of the pyloric rhythm, because in some systems (e.g. zebrafish) the phrase "primary motor neurons" is used to distinguish those neurons from the "secondary motor neurons" which innervate the same muscles but have different synaptic response and intrinsic properties. Done, just removed as unnecessary

8. Line 134/Line 144: These nervous systems were "superfused at a rate of 300-500 ml per day (∼0.2 - 0.4 ml/min)". How does its per minute rate compare to the per minute superfusion rate used during acute experiments?

Done, added description of acute superfusion rates to line 146-148

9. Line 197: Insert "The first component includes" to the start of the sentence that currently begins with "Two PD neurons". Done

10. Line 200: Perhaps "primes activity" is better than "drives activity"?

This is a tough choice but we believe “drives” is better; rebound from inhibition is an important driver of activity in the follower neurons of the rhythm, by activating conductances such as Ih. For example, the LP neuron is commonly silent following decentralization besides bursts which occur shortly following bursts from the PD neuron— thus the inhibition from the pacemaker drives the following burst. Added the word “bursting” to make this process more clear and to distinguish from tonic activity which is sometimes seen without pacemaker drive.

11. Line 204: Add a comma after "pattern generating circuit". Done

12. Line 209: Add "(Fig. 1A)" after "single input nerve, the stn". Done

13. Line 222: Add "either" after "which involved". Done

14. Line 259: Delete "from". Done

15. Line 281: (a) add "positive" between "significant" and "correlation"? Done

(b) The R-value is not provided in the figure; add it here in the text? These are added now to Figure 4. (they are also in the statistical table)

16. Line 284: Another mention of the use of R-values which themselves are not provided in either the text, figure or figure legends?

Added now to Figure 4, and added description in legends. Note p-values of these were also in some cases stated incorrectly in the text (they were correct in the statistical table). This has also been fixed.

17. Line 318: Insert "at least" after "cycles exhibited". Done

18. Line 365: Change to "incubator, and instead of continuous superfusion we changed the saline once per day." Done

19. Lines 374-377: I think that there is an inaccuracy here- Based on Figure 7, it appears that the mean pyloric cycle frequency post-bulk saline exchange is not different from that occurring during continuous perfusion but is significantly different (p<0.001) from that occurring immediately before the bulk exchange. The current text appears to report instead a difference between continuous perfusion and post-bulk exchange but no different pre- vs post-bulk exchange. Fixed.

Sentence had ideas misplaced – the continuously superfused, time-matched examples are not different than pre-exchange frequencies, but are different from post-exchange frequencies.

20. Line 407: How were the crabs determined to be between 5-7 years old? We know they are adults based on size and that it takes approximately so long for them to mature. Precise data for this species are unknown, but they are also heavily fished and probably unlikely to reach a ripe old age. Added a clause about carapace size to address this issue.

21. Lines 410-411: Change "In fact" to "For example". Done

22. Line 411: Insert "the pyloric rhythm in" after "a particularly warm winter,". Done

23. Lines 433-435: Merge references within the same parentheses. Done

24. Lines 438-439: Why is this point interesting? Done – hasn’t been shown before. Thus, the next point –

25. Line 439-440: Has this cycle frequency-dependent distinction between phase-compensated vs not phase-compensated published previously? Is now made explicit.

26. Lines 443-456: What is suggested by the result that cycle frequency increased after bulk saline replacement? That the circuit remains largely unchanged from the control circuit but has a weaker drive due to reduced pacemaker activity caused by oxygen/nutrient starvation, and that the resulting weaker rhythm drives activity-dependent changes to occur in the circuit neurons? Something else? Done, added a long sentence about potential factors.

27. Figures- Nerve Recordings:

(a) It would be helpful to have the extracellular nerve recordings labeled with the acronym of the recorded nerve so that the reader would not need to refer to the figure legend. Done

(b) It would be helpful if each nerve recording in a set (i.e. recordings at different time points) started at the same time point of the rhythm (e.g. a PD neuron burst). Doing so would make it much easier to compare rhythm speed across time points. Done

28. Figure 4B-E: Please define "dc" in the Figure Legend (dc, decentralization). Done

### Reviewer 2:

Although many previous studies have examined the activity of the pyloric circuit after decentralization, this study takes a very thorough quantitative approach with high n values and importantly utilizes continuous long-term recordings instead of sampling at discrete time points. This is a very careful study, the data are novel and very clearly presented and important findings are reported.

The data in this study alter our view of the consequences of removing modulatory inputs. Previous studies suggested cessation of circuit activity that recovered in a periodic fashion (bouting) until arriving at a new solution. This study suggests instead that the more common consequence of removal of modulatory input is a new "normal" which is arrived at much more quickly and then maintained. Interestingly, the new solution has both similarities and differences to circuit output in the presence of modulatory inputs. The authors also point that that comparisons of these data to previous data sets, to the extent possible, highlight complications that can arise from using wild-caught research animals, an important note for the scientific community. Additionally, comparing different measures used in previous studies to define "recovery" illustrated that these are actually not measuring the same thing which is important that the field be made aware of this for future consideration of both previous and future data. I have only minor comments.

Minor comments:

1. lines 261-263. Figure 3G is apparently mis-referenced as being both an example of a steady, lower-frequency rhythm throughout as well as exhibiting wide variation on short-time scales.

Fixed, removed as example of variable behavior for clarity.

2. Line 350. I believe this should actually be referencing Fig. 4 which contains the plots of CV and cycle to cycle variability. Figure 5 is intended; the increases in variability over time in Figure 4 are not significant. However the increases in bouting activity over time are – this is the consistent observation intended. Changed “variability” to “bouting” to reflect this.

3. Lines376-378. "....post-exchange frequencies were significantly increased relative to continuously superfused preparations, but not significantly different than frequencies prior to saline exchanges." Aren't the post-exchange frequencies in fact different from those prior to saline exchanges?

Done, fixed now as the sentence was improperly written. Same issue as (19) from reviewer one.

4. Line 433. Include Tang et al 2010 within same parenthesis set as the rest of the references which follow. Done

5. Line 638. Delete "after" at the end of the line. Done

6. Line 663. There is no arrow in (A), only in (B). Fixed in legend.

7. Line 677. Delete "to". Done
